# Bidirectional Hypoxic Extracellular Vesicle Signaling Between Müller Glia and Retinal Pigment Epithelium Regulates Retinal Metabolism and Barrier Function

**DOI:** 10.3390/biology14081014

**Published:** 2025-08-07

**Authors:** Alaa M. Mansour, Mohamed S. Gad, Samar Habib, Khaled Elmasry

**Affiliations:** 1 Department of Oral Biology, The Dental College of Georgia, Augusta University, Augusta, GA 30912, USA; alaa_mamdouh@mans.edu.eg (A.M.M.); mgad@augusta.edu (M.S.G.); dr_samarhabib@mans.edu.eg (S.H.); 2 DCG Center for Excellence in Research, Scholarship, and Innovation (CERSI), Augusta University, Augusta, GA 30912, USA; 3 Department of Oral Biology, Faculty of Dentistry, Mansoura University, Mansoura 35516, Egypt; 4 Department of Medical Histology and Cell Biology, Faculty of Medicine, Mansoura University, Mansoura 35516, Egypt; 5 Department of Medical Parasitology, Faculty of Medicine, Mansoura University, Mansoura 35516, Egypt; 6 Department of Cellular Biology and Anatomy, The Medical College of Georgia, Augusta University, Augusta, GA 30912, USA; 7 Department of Human Anatomy and Embryology, Faculty of Medicine, Mansoura University, Mansoura 35516, Egypt

**Keywords:** Müller glia, retinal pigment epithelium, extracellular vesicles, exosomes, proteomics, ECIS, GFAP, blood–retinal barrier, hypoxia, retinopathy

## Abstract

The retina is highly sensitive to oxygen and blood supply, and hypoxia plays a key role in retinal diseases such as diabetic retinopathy (DR) and age-related macular degeneration (AMD). Müller glial cells, which are essential for retinal homeostasis, respond to injury and hypoxia with reactive gliosis, characterized by the upregulation of the glial fibrillary acidic protein (GFAP) and vimentin, cellular hypertrophy, and extracellular matrix changes, which can impair retinal function and repair. The retinal pigment epithelium (RPE) supports photoreceptors, forms part of the blood–retinal barrier, and protects against oxidative stress; its dysfunction contributes to retinal degenerative diseases such as AMD, retinitis pigmentosa (RP), and Stargardt disease (SD). Extracellular vesicles (EVs) play a crucial role in intercellular communication, protein homeostasis, and immune modulation, and have emerged as promising diagnostic and therapeutic tools. Understanding the role of extracellular vesicles’ (EVs’) signaling machinery of glial cells and the retinal pigment epithelium (RPE) is critical for developing effective treatments for retinal degeneration. In this study, we investigated the bidirectional EV-mediated crosstalk between RPE and Müller cells under hypoxic conditions and its impact on cellular metabolism and retinal cell integrity. Our findings demonstrate that RPE-derived extracellular vesicles (RPE EVs) induce time-dependent metabolic reprogramming in Müller cells. Short-term exposure (24 h) promotes pathways supporting neurotransmitter cycling, calcium and mineral absorption, and glutamate metabolism, while prolonged exposure (72 h) shifts Müller cell metabolism toward enhanced mitochondrial function and ATP production. Conversely, Müller cell-derived EVs under hypoxia influenced RPE metabolic pathways, enhancing fatty acid metabolism, intracellular vesicular trafficking, and the biosynthesis of mitochondrial co-factors such as ubiquinone. Proteomic analysis revealed significant modulation of key regulatory proteins. In Müller cells, hypoxic RPE-EV exposure led to reduced expression of Dyskerin Pseudouridine Synthase 1 (DKc1), Eukaryotic Translation Termination Factor 1 (ETF1), and Protein Ser/Thr phosphatases (PPP2R1B), suggesting alterations in RNA processing, translational fidelity, and signaling. RPE cells exposed to hypoxic Müller cell EVs exhibited elevated Ribosome-binding protein 1 (RRBP1), RAC1/2, and Guanine Nucleotide-Binding Protein G(i) Subunit Alpha-1 (GNAI1), supporting enhanced endoplasmic reticulum (ER) function and cytoskeletal remodeling. Functional assays also revealed the compromised barrier integrity of the outer blood–retinal barrier (oBRB) under hypoxic co-culture conditions. These results underscore the adaptive but time-sensitive nature of retinal cell communication via EVs in response to hypoxia. Targeting this crosstalk may offer novel therapeutic strategies to preserve retinal structure and function in ischemic retinopathies.

## Simple Summary

The retina’s sensitivity to oxygen makes it highly susceptible to hypoxia, contributing to diseases like diabetic retinopathy and macular degeneration. Müller glial cells respond with reactive gliosis, while RPE dysfunction disrupts retinal integrity. This study shows that under hypoxia, extracellular vesicles (EVs) mediate bidirectional communication between Müller cells and the RPE, driving time-dependent metabolic reprogramming. RPE EVs initially enhance neurotransmitter cycling in Müller cells, later shifting toward mitochondrial activation, while hypoxic Müller EVs promote lipid metabolism and ER function in the RPE. Proteomic changes and compromised barrier integrity highlight EVs as key modulators of retinal response to hypoxia and potential therapeutic targets.

## 1. Introduction

The retina is a metabolically active tissue that is extremely sensitive to blood and oxygen perfusion. Any altered blood supply to the eye affects the retina. Systemic hypoxemia (lung or heart disease) or a local vascular disease in the retina can cause retinal hypoxia. All animal cells need oxygen for ATP production to fuel metabolic reactions, and even the smallest changes in oxygen tension bring about adjustments to maintain oxygen homeostasis. However, there are specific retinal diseases in which low oxygen tension either contributes to or plays a crucial role in new vessel formation, such as diabetic retinopathy (DR) and age-related macular degeneration (AMD) [1].

Müller cells are the predominant class of glial cells in the vertebrate retina. They span the entire width of the retina from the inner to the outer limiting membrane. Additionally, they are intimately involved in the visual cycle through synthesizing and renewing cone visual pigments, providing anti-oxidative support for neurons and photoreceptors, regulating the tightness of the blood–retinal barrier (BRB), maintaining the normal transmitter milieu, and controlling calcium and water homeostasis within the retina [2].

Glial fibrillary acidic protein (GFAP) is a member of the intermediate filament family of proteins, a group that contributes to the formation of the cell cytoskeleton that is normally expressed by Müller cells at low levels but becomes upregulated in response to retinal injury, neuronal degeneration, retinal detachment, light damage, or mechanical lesioning. Additional responses of Müller cells to injury include hypertrophy, proliferation, migration, and altered expression of enzymes and intermediate filament proteins [3].

Gliosis is a reactive process in the central nervous system (CNS), including the brain, spinal cord, and retina, that occurs in response to injury, disease, or stress. It involves the activation and proliferation of certain glial cells, including Astrocytes in the CNS and Müller cells in the retina. It involves increased expression of intermediate filament proteins, especially GFAP and vimentin, along with glial cell enlargement (hypertrophy) and the buildup of inhibitory extracellular matrix components. This process can hinder tissue function and repair. Although the exact role of intermediate filaments in gliosis is debated, they are thought to help maintain the structural changes in glial cells. Reactive gliosis specifically features elevated levels of these proteins and morphological changes in glial cells, such as process thickening and stabilization [4].

Ischemic retinopathies often involve neuronal loss and abnormal blood vessel growth. Müller glial cells in the retina produce pigment epithelium-derived factor (PEDF), a neuroprotective and anti-angiogenic protein that helps counteract elevated vascular endothelial growth factor (VEGF) levels [5], which are mainly regulated by hypoxia-inducible factor 1 (HIF-1) during hypoxia [6]. In hypoxic conditions, these cells also increase vascular permeability, contributing to disease progression [7,8]. Modulating Müller glial behavior—by inhibiting gliosis and encouraging reprogramming—offers a promising strategy for treating retinal degeneration [9].

The retinal pigment epithelium (RPE) consists of a single layer of uniformly shaped polygonal cells located at the retina’s outermost region. Its outer surface interfaces with Bruch’s membrane and the choroid, while the inner surface contacts the photoreceptor outer segments. Basal infoldings on the outer side enhance the cell’s surface area, promoting the efficient exchange of substances. The basement membrane is tightly linked to these basal folds through half-desmosomes found in the innermost part of Bruch’s membrane [10].

Tight junctions between the single-layered RPE and adjacent gap junctions regulate substance movement and, together with Bruch’s membrane and the choroid, form the choroid–blood–retinal barrier along the peripheral retina [11]. The RPE also contains a sophisticated metabolic system that mitigates the buildup of reactive oxygen species (ROS), protecting against oxidative damage [12]. Because of its critical structural and functional roles, any disruption in the RPE can compromise vision and contribute to retinal diseases such as retinitis pigmentosa (RP), age-related macular degeneration (AMD), and Stargardt disease (SD) [13].

Extracellular vesicles (EVs) are small, single-membrane vesicles ranging from about 30 to 200 nanometers in diameter. They share the same membrane orientation as their parent cells and are rich in specific proteins, lipids, nucleic acids, and glycoconjugates. These vesicles contain complex, membrane-bound protein structures and exhibit significant molecular diversity [14].

EVs are formed through budding from the plasma membrane or within endosomes and play a key role in cellular protein quality control. Once released, they participate in various functions, including extracellular matrix remodeling and intercellular communication [15]. EVs are also involved in numerous physiological and pathological processes, such as development, immune responses, tissue maintenance, cancer, and neurodegenerative diseases. Due to these versatile roles, EVs are being explored as potential diagnostic and therapeutic tools across a range of disorders [16,17,18].

The current study suggests that under hypoxic conditions, EVs derived from RPE cells mediate metabolic reprogramming in Müller glial cells. Conversely, Müller cell-derived EVs under hypoxia may influence RPE cell metabolism and barrier function, suggesting a bidirectional EV-mediated crosstalk that helps maintain retinal homeostasis and structural integrity during metabolic stress.

## 2. Materials and Methods

### 2.1. Retinal Pigment (RPE) and Müller Cell (RMC) Culture

Immortalized Rat Müller cells (RMCs) were purchased from Applied Biological Materials Inc. (Richmond, BC, Canada) (abm, USA Catalog # T0576). They were cultured with DMEM-F12 (Dulbecco’s Modified Eagle Medium/Nutrient Mixture F-12, Thermo Fischer Scientific, Waltham, MA, USA, Catalog # 11320033). The Immortalized ARPE-19 cells were purchased from the American Type Culture Collection (Manassas, VA, USA) (ATCC, Catalog # CRL-2302). According to the source recommendation, they were cultured with DMEM-F12 (Dulbecco’s Modified Eagle Medium/Nutrient Mixture F-12, ATCC, USA Catalog # 302006) containing 10% fetal bovine serum (FBS) (Gibco™ Fetal Bovine Serum, Fischer Scientific, Waltham, MA, USA, Catalog # A5670801). Both RMCs and ARPE-19 were incubated—passages (3–8), put into 75 t flasks in 10 mL of media till 80–90% confluency, under both normoxia and hypoxia conditions. The normoxia condition was performed by incubating the cells at 37 °C, 5% CO_2_. The hypoxia condition was established by incubating the cells in a hypoxia chamber at 37 °C, 5% CO_2_, and 2.5% O_2_.

### 2.2. Isolation of Extracellular Vesicles (EVs)

EVs were isolated using total exosomes’ isolation reagent (Invitrogen, Thermo Fisher Scientific, Waltham, MA, USA, Catalog # 4478359) from cell culture medium harvested after 24 h, 48 h, and 72 h of incubation following the manufacturer’s protocol. Collected media were centrifuged for 30 min at 2000× *g* to remove cell debris. The supernatant was then transferred to a new tube and mixed with the reagent at a 2:1 ratio, then left at 4 °C overnight. Samples were recentrifuged at 10,000× *g* at 4 °C for 65 min. The final pellets were resuspended in 1 × phosphate-buffered saline (PBS). The isolated EVs were stored at 2 °C to 8 °C for up to 1 week, or at ≤20 °C for long-term storage.

### 2.3. EV Characterizations

#### 2.3.1. Nanoparticle Tracking of the Isolated Extracellular Vesicles

The isolated EVs were studied with ZetaView@ (Particle Matrix Inc., Wildmoos, Inning am Ammersee, Germany) using both scatter and fluorescence mode (488 nm laser, 40 mW power) for information about the average size and concentration/mL of EVs in the collected samples.

#### 2.3.2. Transmission Electron Microscope (TEM)

To confirm the existence of EVs in the sample, a life science TEM (JOEL JEM-1220, Tokyo, Japan) was used. A total of 10 μL of a 1-in-10 dilution of the samples was applied to formvar/carbon-coated nickel TEM grids, and they were left incubating for 30 min. Then, deionized water was used to wash the grids twice. After that, the grids were dried and stained with phosphotungstic acid [19]. In addition, Immunogold labeling of EVs was performed using Anti-CD63 (Thermo Fischer, Waltham, MA, USA, Catalog # PA5-100713). Grids were examined in a JEM 1400 Flash TEM (JEOL USA Inc., Peabody, MA, USA) at 110 kV and imaged with a Gatan One View Digital Camera (Gatan Inc., Pleasanton, CA, USA).

### 2.4. Assessment of the Effect of Co-Culturing RMCs and RPE EVs

#### 2.4.1. GFAP Immunofluorescence (IF) for RMCs

Müller cells were seeded in Falcon cell culture slides in 8 chambers and cultured with RPE EVs for 24 and 72 h. The concentrations for EVs were 160 × 10^6^ particles/µL. The cells were fixed for IF. Therefore, there were 7 groups in each period as follows:

Group I: 4 × 10^4^ RMCs cultured in complete culture media without any addition.

Group II: 4 × 10^4^ RMCs co-cultured with RPE EVs isolated under normoxia after 24 h.

Group III: 4 × 10^4^ RMCs co-cultured with RPE EVs isolated under normoxia after 48 h.

Group IV: 4 × 10^4^ RMCs co-cultured with RPE EVs isolated under normoxia after 72 h.

Group V: 4 × 10^4^ RMCs co-cultured with RPE EVs isolated under hypoxia after 24 h.

Group VI: 4 × 10^4^ RMCs co-cultured with RPE EVs isolated under hypoxia after 48 h.

Group VII: 4 × 10^4^ RMCs co-cultured with RPE EVs isolated under hypoxia after 72 h.

For IF Staining, the cells were fixed using 10% formaldehyde for 10 min. The antigen retrieval was performed using an antigen retrieval solution. The slides were placed in the blocking buffer (BSA, goat serum, and TritonX mixture) for at least 30 min. After quick washes using PBS, ×1, the GFAP Primary antibody (catalog number PA1-10019) was added to the slides and left for 2 h or overnight at RT or 4 degrees with blocking buffer in a ratio of 1:250. The next day, slides were washed in PBS ×2. Secondary Antibody tagged with green fluorescence was added to the slides. Then, slides were washed by ×2 PBS. The coverslip was placed using Vectashield DAPI mounting media (Sigma-Aldrich Chemical Corp., St. Louis, MO, USA). The slides were stored and examined in the dark.

#### 2.4.2. Proteomics for RMCs

RMCs were seeded in a 6-well plate and cultured with RPE EVs for 24 and 72 h. The concentrations for EVs were 160 × 10^6^ particles/µL. Then, the cells were harvested and collected in Eppendorf. The cells were divided into the following groups:

Group I: RMCs normally cultured in complete culture media.

Group II: RMCs co-cultured with RPE EVs isolated under normoxia after 24 h.

Group III: RMCs co-cultured with RPE EVs isolated under normoxia after 72 h.

Group IV: RMCs co-cultured with RPE EVs isolated under hypoxia after 24 h.

Group V: RMCs co-cultured with RPE EVs isolated under hypoxia after 72 h.

LC-MS/MS Sample Preparation and Analysis Summary.

Cell Lysis: Cells were harvested using 0.25% EDTA-trypsin, centrifuged at 1200 rpm for 5 min, and washed with PBS. The cell pellets were lysed using RIPA buffer and centrifuged. The resulting supernatant was collected, and protein concentration was quantified using the BCA assay.

Protein Digestion: Trypsin was added at a 1:25 enzyme-to-protein ratio, and digestion proceeded at 37 °C for 16 h. The peptides were collected via centrifugation, eluted with ddH_2_O and NH_4_HCO_3_, and dried at 45 °C.

Data Acquisition: Peptide samples were analyzed using the Easy-nLC 1200 system coupled with an Orbitrap Fusion Lumos mass spectrometer in Data-Dependent Acquisition (DDA) mode. Data was acquired in real-time using XCalibur 4.3 software.

Data Analysis: Raw MS data were analyzed using MaxQuant (v2.0.3.0), referencing the Human Uniprot database. The FDR threshold was <1%. Protein expression data from both RMC and RPE cells were normalized and analyzed statistically using Perseus (v2.0.3.0), and pathway enrichment was conducted using Metascape (https://metascape.org/gp/index.html#/main/step1, accessed on 4 August 2025) with KEGG, Reactome, and GO databases.

Data Normalization: Targeted proteomics data were normalized against reference proteins, which were GAPDH and β-actin. Therefore, the target protein level was normalized by dividing it by the normalization factor. The normalization factor was the mean of both GAPDH and β-actin levels.

### 2.5. Assessment of the Effect of Co-Culturing RPE and RMC EVs

#### 2.5.1. Electric Cell-Substrate Impedance Sensing Method (ECIS)

The electric cell-substrate impedance sensing [ECIS^®^Zθ (theta)] instrument (Applied Biophysics Inc., Troy, NY, USA) was used to measure the normalized transcellular electrical resistance (TER) of the RPE as an indicator of the barrier integrity. Briefly, electrode arrays (96-wells, Applied Biophysics Inc., Catalog # 96W20idf PET) coated with 0.02% gelatine and 100 μM of cysteine were used to culture RPE. The cells were either cultured alone or co-cultured with 80 × 10^6^ particles/µL of RMC EVs isolated after 24 h, 48 h, or 72 h under both normoxia and hypoxia conditions. Accordingly, the RPE was also divided into 7 groups. TER was independently measured in each well over the experimental time course (every 24 h). Plotting of the normalized resistance as a function of time was performed after calculating the ratio of the measured resistance to baseline resistance.

#### 2.5.2. Proteomics for RPE

RPEs were seeded on a 6-well plate and cultured with RMC EVs for 24 and 72 h. The concentrations for EVs were 160 × 10^6^ particles/µL. Then, the cells were harvested and collected in Eppendorf. The groups were as follows:

Group I: RPEs cultured alone in complete culture media.

Group II: RPEs co-cultured with RMC EVs isolated under normoxia after 24 h.

Group III: RPEs co-cultured with RMC EVs isolated under normoxia after 72 h.

Group IV: RPEs co-cultured with RMC EVs isolated under hypoxia after 24 h.

Group V: RPEs co-cultured with RMC EVs isolated under hypoxia after 72 h.

The remaining steps for LC-MS/MS Sample Preparation and Analysis are the same as mentioned previously with RMC proteomics.

### 2.6. Statistical Analysis

The results are demonstrated as means ± SEM. One-way ANOVA or a two-tailed *t*-test was used to differentiate between different experimental groups. Results were considered significant for *p* < 0.05.

## 3. Results

### 3.1. EV Characterization

#### 3.1.1. Nanoparticle Tracking Analysis by Zetaveiw Analysis

**Figure 1:**
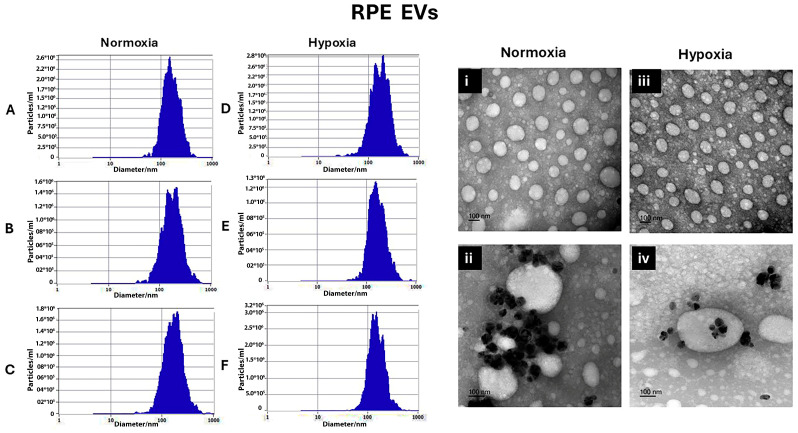
Characterization of RMC EVs by showing the normoxia RPE EV sizes after 24 h, 48 h, and 72 h, represented using Zetaveiw nanoparticle tracking, respectively (**A**–**C**), and the shape and immunogold labeling of isolated EVs using EM imaging (**i**,**ii**). Similarly, for the hypoxia RPE EVs, the sizes after 24 h, 48 h, and 72 h were represented in (**D**–**F**) and their shape and immunogold labeling are represented in (**iii**,**iv**).

The normoxic RPE EV sizes were 168.3 ± 13.11 nm, 194.4 ± 14.3 nm, and 185.14 ± 9 nm, after 24 h, 48 h, and 72 h, respectively, as shown in Figure 1A–C. On the other hand, hypoxia EV sizes for 24 h, 48 h, and 72 h were 163.9 ± 20.7 nm, 167.4 ± 10.6 nm, and 164.5 ± 12.6 nm, respectively, as shown in Figure 1D–F.

**Figure 2:**
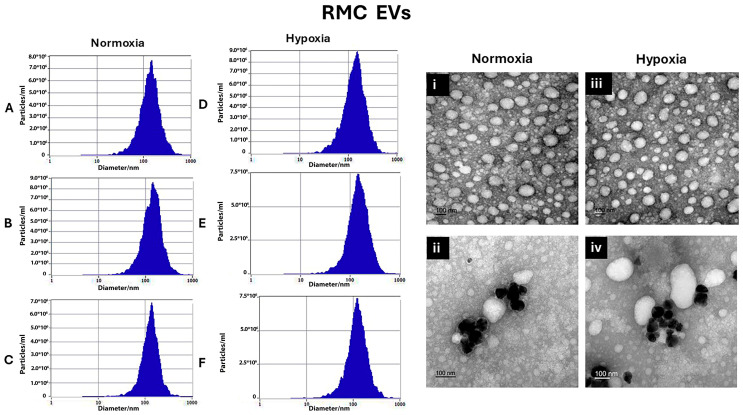
Characterization of RMC EVs by showing the sizes of the normoxia-induced RMC EVs after 24 h, 48 h, and 72 h, represented using Zetaveiw nanoparticle tracking, respectively (**A**–**C**), and the shape and immunogold labeling of isolated EVs using EM imaging (**i**,**ii**). Similarly, for the hypoxia RPE EVs, the sizes after 24 h, 48 h, and 72 h are represented in (**D**–**F**), and their shape and immunogold labeling are shown in (**iii**,**iv**).

For RMC EVs, normoxic RMC EVs sizes were 154.8 ± 19.9 nm, 150.45 ± 16.2 nm, and 148.9 ± 286 nm, respectively. On the other hand, hypoxia RMC EVs sizes for 24 h, 48 h, and 72 h were 148.02 ± 12.1 nm, 153.4 ± 9.4 nm, and 152.8 ± 20.2 nm, respectively, as shown in Figure 2A–C and Figure 2D–F.

#### 3.1.2. Transmission Electron Microscope

Transmission electron microscopy was used to confirm the presence of EVs in the suspensions and can be used to describe EVs’ morphology and match the size that was found by Zetveiw analysis. EV shapes were oval or spherical. Anti-CD63, immunogold labeling detected CD-63 inside and on the surface of the EVs. (RPE EVs: Figure 1i,ii) (RMC EVs: Figure 2i,ii)

### 3.2. Co-Culture RMCs and RPE EVs

#### 3.2.1. Time-Dependent GFAP Upregulation in RMCs Treated with Hypoxic and Normoxic RPE EVs

**Figure 3:**
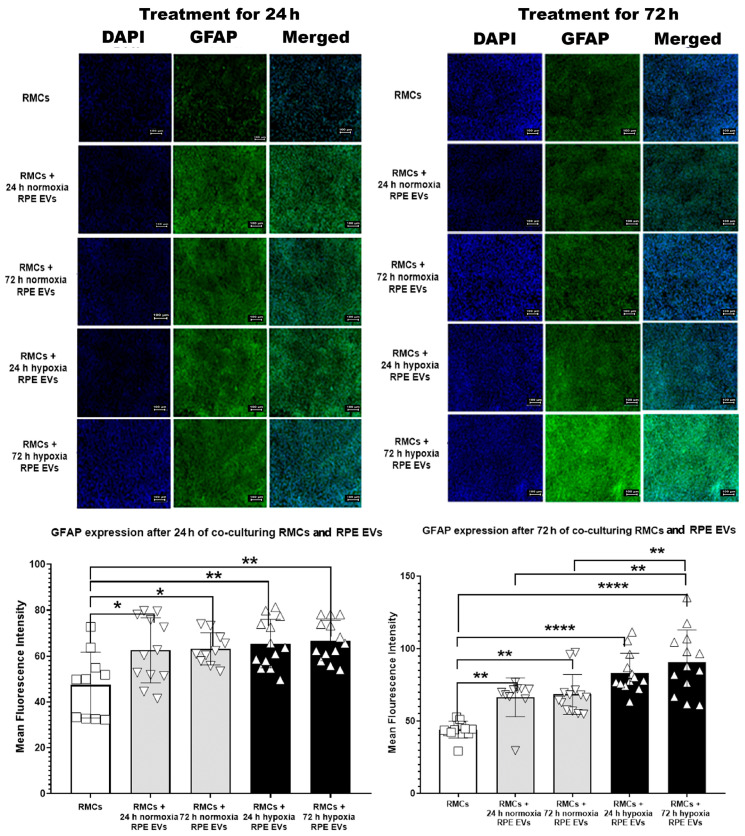
Time-Dependent GFAP Upregulation in RMCs Treated with Hypoxic and Normoxic RPE EVs. This reactivity after 24 and 72 h was less noted in the RMC control group. GFAP immune-expression after 24 h of incubation was significantly higher in the RPE EV-treated groups—either normoxic or hypoxic—than in the control group. Meanwhile, there was a non-significant difference between the EV-treated groups. After 72 h of RMC and RPE EV co-culturing, GFAP also showed a marked rise in RPE EV-treated groups, but it was more significant in RMCs treated with 72 h hypoxic RPE EVs. GFAP expression was also significantly higher in RMCs treated with 72 h hypoxic RPE EVs when compared to both RMC groups treated with either 24 h or 72 h normoxic RPE EVs. Data represent individual data points with means ± SEM; *n* = 13/group; * *p* < 0.05; ** *p* < 0.01; **** *p* < 0.0001. Scale bar: 100 µm.

As anti-GFAP is a widely used marker for retinal injury and/or reactive Müller glial cells and gliosis, it has been selected to detect the effect of RPE EVs isolated after a hypoxic condition versus a normal condition. This reactivity after 24 and 72 h was less noted in the RMC control group. GFAP immune-expression after 24 h of incubation was significantly higher in the RPE EV-treated groups—either normoxic or hypoxic—than in the control group. Meanwhile, there was a non-significant difference between the EV-treated groups. After 72 h of RMC and RPE EV co-culturing, GFAP also showed a marked rise in RPE EV-treated groups, but it was more significant in RMCs treated with 72 h of hypoxic RPE EVs. GFAP expression was also significantly higher in RMCs treated with 72 h hypoxic RPE EVs when compared to both RMC groups treated with either 24 h or 72 h normoxic RPE EVs, as shown in Figure 3. This significant change can be attributed to either hypoxia and/or the duration of treatment.

#### 3.2.2. Proteomic Profiling Reveals Temporal Pathway Modulation in RMCs by Hypoxia-Induced RPE EVs

**Figure 4:**
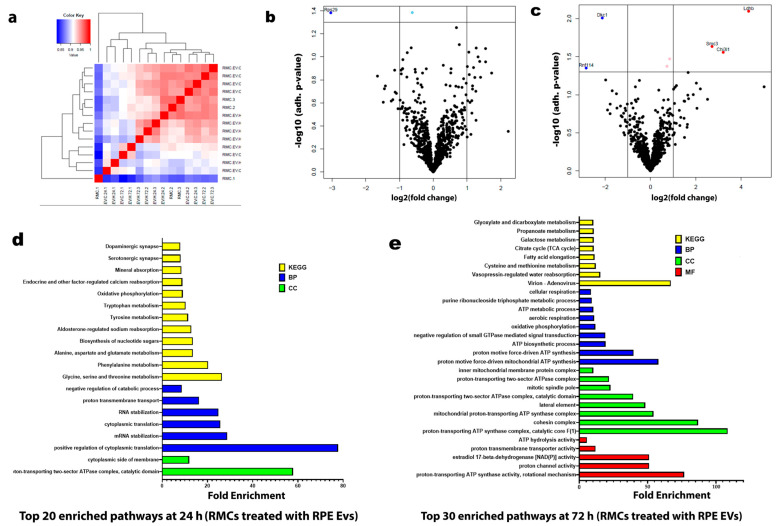
Proteomic Profiling Reveals Temporal Pathway Modulation in RMCs by Hypoxia-Induced RPE EVs. (**a**) Hierarchical clustering heatmap of the proteomic expression of the RMC studied groups. (**b**) Volcano map of distinguishable protein expression profiling in RMCs treated with hypoxic RPE EVs for 24 h. (**c**) Volcano map of distinguishable protein expression profiling in RMCs treated with hypoxic RPE EVs for 72 h. (**d**) List of the top 20 enriched pathways in RMCs treated with hypoxic RPE EVs for 24 h. (**e**) List of the top 30 enriched pathways in RMCs treated with hypoxic RPE EVs for 72 h. The top basic functions were categorized into the following classes: molecular function (MF) (Red), cellular component (CC) (Green), biological process (BP) (Blue), and Kyoto Encyclopedia of Genes and Genomes (KEGG) (Yellow).

Under hypoxia, there were notable changes after the analysis of the proteomic data and the analysis of the RMC protein enrichment after co-culturing with RPE EVs for 24 h and 72 h. After 24 h of co-culturing, the top 20 enriched pathways were correlated to cytoplasmic translation processes, RNA stabilization, amino acid metabolism, mineral absorption, and synaptic functions. Meanwhile, after 72 h of co-culturing, the top 30 enriched pathways were mainly related to mitochondrial activity and ATP-related processes, including ATP synthase, oxidative phosphorylation, aerobic respiration, and citric cycle. (Figure 4).

### 3.3. Co-Culture RPE and RMC EVs

#### 3.3.1. Hypoxic RMC EVs Disrupt RPE Barrier Integrity in a Time-Dependent Manner

**Figure 5:**
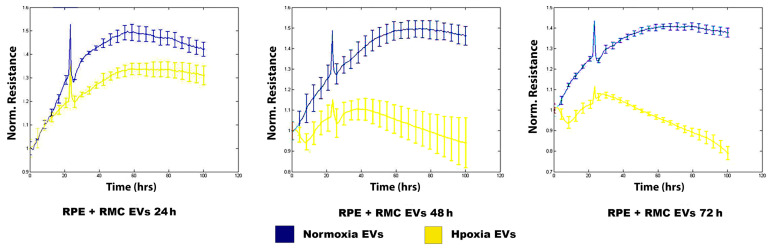
Effect of normoxic and hypoxic RMC EVs on RPE barrier function. ECIS analysis demonstrated a marked decrease in the TER of the RPE treated with hypoxia RMC EVs compared over time (yellow lines) to those treated with normoxia RMC EVs (blue lines). Data points represent the mean of 4–6 wells at the same time points. Data represent individual data points with means ± SEM; *n* = 5/group.

The integrity of junctions between the RPE cells treated with normoxia and hypoxia RMC EVs for 24 h, 48 h, and 72 h was assessed by ECIS which demonstrated a non-significant change in the intercellular resistance among the RPE cells treated with normoxia RMC EVs for 24 h, 48 h, and 72 h (blue line). An evident decrease in TER was detected in RPEs co-cultured with hypoxic RMC EVs for 24 h, and this attenuation progressed over time after 48 h and 72 h of co-culturing with hypoxic RMC EVs (yellow line) (Figure 5).

#### 3.3.2. Hypoxic RMC EVs Induce Distinct Temporal Proteomic Signatures in RPE Cells

**Figure 6:**
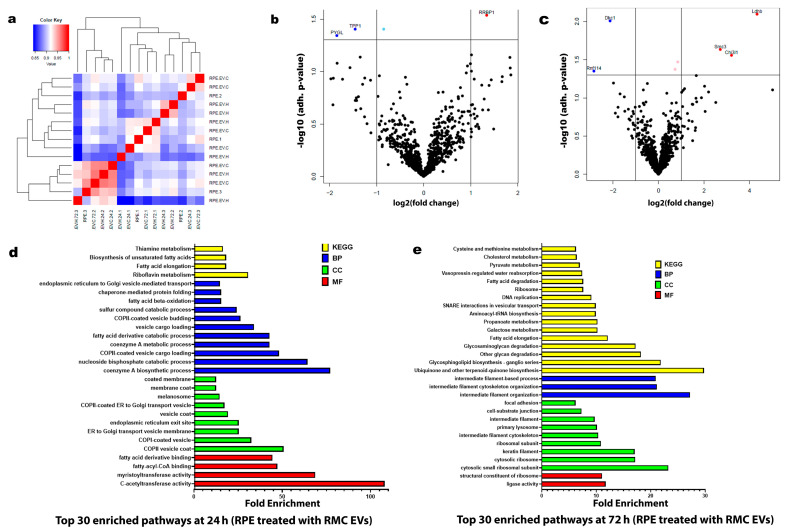
Proteomic profiles of the RPE treated with normoxic and/or hypoxic RMC EVs. (**a**) Hierarchical clustering heatmap of the proteomic expression of the RPE studied groups. (**b**) Volcano map of a distinguishable protein expression profiling in RPEs treated with hypoxic RMC EVs for 24 h. (**c**) Volcano map of a distinguishable protein expression profiling in RPEs treated with hypoxic RMC EVs for 72 h. (**d**) List of the top 30 enriched pathways in RPEs treated with hypoxic RMC EVs for 24 h. (**e**) List of the top 30 enriched pathways in RPEs treated with hypoxic RMC EVs for 72 h. The top basic functions were categorized into the following classes: molecular function (MF) (Red), cellular component (CC) (Green), biological process (BP) (Blue), and Kyoto Encyclopedia of Genes and Genomes (KEGG) (Yellow).

Under hypoxia, there were notable changes after the analysis of the proteomic data and the analysis of the RPE protein enrichment after co-culturing with RMC EVs for 24 h and 72 h. After 24 h of co-culturing, the top 30 enriched pathways were correlated to ER activity and cellular vesicular transport, together with fatty acid metabolism. Meanwhile, after 72 h of co-culturing, the top 30 enriched pathways were mainly related to biosynthetic pathways, including protein synthesis, cytoskeletal protein formation, and cellular support and adhesion (see Figure 6).

### 3.4. Proteomic Validation Results

In validating the proteomic results for RMCs treated with RPE EVs, results showed a significant downregulation of DKc1, Etf1, and PPP2R1B protein expression in RMCs treated with RPE EVs after 72 h of hypoxia compared to those RMCs treated with RPE EVs under normoxia for 24 h.

Upon validating the proteomic results for RPEs treated with RMC EVs, we found the following: Upon comparing the protein expressions in RPEs treated with RMC EVs after 24 h of hypoxia, those RPEs treated with RMC EVs under normoxia for 24 h showed a significant elevation of RRBP1, RAC1, RAC2, and GNAI1. On the other hand, there was a non-significant rise in ATP5F1D expression. BLVRB gene expression was significantly lower in RPEs treated with RMC EVs after 72 h of hypoxia when compared to RPEs treated with RMC EVs under normoxia for 24 h.

## 4. Discussion

The retina ranks among the most metabolically active organs in the human body. Its metabolic processes are tightly regulated to ensure an adequate supply of blood and oxygen that is necessary for the neural transmission of visual signals [20]. Two vascular systems support the retina: the choriocapillaris and the central retinal artery. The choriocapillaris nourishes the outer layers of the retina, including the RPE and photoreceptors, while the central retinal artery delivers oxygen to the inner retina, including the retinal ganglion cells and the retinal nerve fiber layers [21,22]. Retinal arteries are regulated by vasogenic factors released from the endothelium in response to intraocular pressure and the retina’s metabolic demands [23].

The retina meets its energy demands primarily through aerobic respiration and β-oxidation. However, during metabolic stress or low-oxygen conditions (hypoxia), it increasingly depends on alternative pathways like the pentose phosphate pathway and anaerobic metabolism [24]. Notably, hypoxia has also been shown to enhance β-oxidation, suggesting that this process plays a role in both healthy and diseased states. These metabolic shifts help retinal cells adapt and survive under hypoxic conditions [25].

As mentioned before, the RPE supports the retina through maintaining retinal structure and function, conducting phagocytosis, forming the outer BRB, transporting nutrients, ions, and water, absorbing light, shielding retinal cells from photo-oxidative damage, sustaining the visual cycle, and producing essential factors. Therefore, it is essential for proper eye function, and any disruptions in this layer structurally or functionally cause several eye diseases [10,26]. Disruptions in the structure or function of the RPE are closely linked to several eye diseases, such as age-related macular degeneration (AMD), proliferative vitreoretinopathy (PVR), Stargardt disease, retinitis pigmentosa (RP), and diabetic retinopathy (DR) [13].

Müller cells are the principal radial glial cells of the retina, extending across its full thickness and interacting with all types of retinal cells [27]. In disease states, various intracellular signaling pathways become activated within Müller cells, leading to the increased production of pro-angiogenic factors and the suppression of anti-angiogenic factors. These responses are further intensified by the proliferation and dedifferentiation of Müller cells [28].

Extracellular vesicles (EVs) facilitate intercellular communication by transferring bioactive molecules such as proteins, lipids, mRNAs, and microRNAs. In the retina, especially under stress conditions like hypoxia, EVs could play a vital role in mediating the crosstalk between Müller glial cells and RPE cells—two cell types that are crucial for maintaining retinal structure, homeostasis, and function [29,30,31]. Under hypoxic conditions, both Müller cells and RPE cells undergo metabolic and functional alterations. Hypoxia can stimulate the release of EVs with altered cargo, reflecting the cellular stress response [32,33].

In the current work, culturing RMCs together with RPE EVs, the GFAP expression was evaluated by IF as GFAP is a widely used marker for retinal injury (Figure 3). Upregulation of GFAP in Müller cells indicates a reactive state within the retina, often in response to injury or disease. These glial cells play a dynamic role in modulating inflammation, contributing to both pro- and anti-inflammatory processes that shape the retinal immune landscape. Understanding the relationship between GFAP expression and the release of inflammatory mediators is essential for advancing targeted therapeutic strategies for retinal disorders [34,35]. GFAP expression showed significant elevation under both normoxic and hypoxic conditions. GFAP can initially help stabilize the retinal environment. During hypoxia, it undergoes upregulation due to Müller cell activation and retinal stress. Prolonged or excessive GFAP expression may contribute to pathological remodeling and visual impairment [36]. GFAP expression during hypoxia may be influenced by HIF-1α signaling, a key transcription factor activated under low-oxygen conditions [37].

Using pathway enrichment analysis of the proteomics data, we investigated the underlying metabolic pathways that may be changed between normoxia and hypoxia during the co-culturing of RMCs with RPE EVs (Figure 4). After 24 h of culturing RMCs with hypoxic RPE EVs, the enriched pathways including the cellular components, biological processes, and genomic expression were mainly directed to maintain the normal function of RMCs including cytoplasmic protein formation, RNA stabilization, glutamate and aspartate metabolism, and calcium and mineral absorption in addition to normal neurotransmitter function such as that of dopamine and serotonin (Figure 4d). [27]. Glutamate, a neurotransmitter released by neurons, is taken up by Müller cells through the glutamate-aspartate transporter. Within Müller cells, the reclaimed glutamate is converted into glutamine by the enzyme glutamine synthetase, which is abundantly expressed in these cells. The resulting glutamine is then released by Müller cells and transported back to neurons for reuse [38].

On the other hand, after culturing RMCs with hypoxic RPE EVs for 72 h, metabolic reprogramming occurred, and the enriched pathways were aligned to enhanced mitochondrial function and ATP production, including enhanced proton-transporting ATP synthase, negative control of small GTPase, enhanced oxidative phosphorylation, and the tricarboxylic acid cycle (TCA) (Figure 4e). These findings are in agreement with previous studies that indicated that normally, ATP in Müller cells is mainly obtained through the glycolytic pathway, and the oxygen consumption is extremely low. Thus, Müller cells tolerate persistent hypoxia and hypoglycemia, saving oxygen and providing energy for the neurons [39].

**Figure 7:**
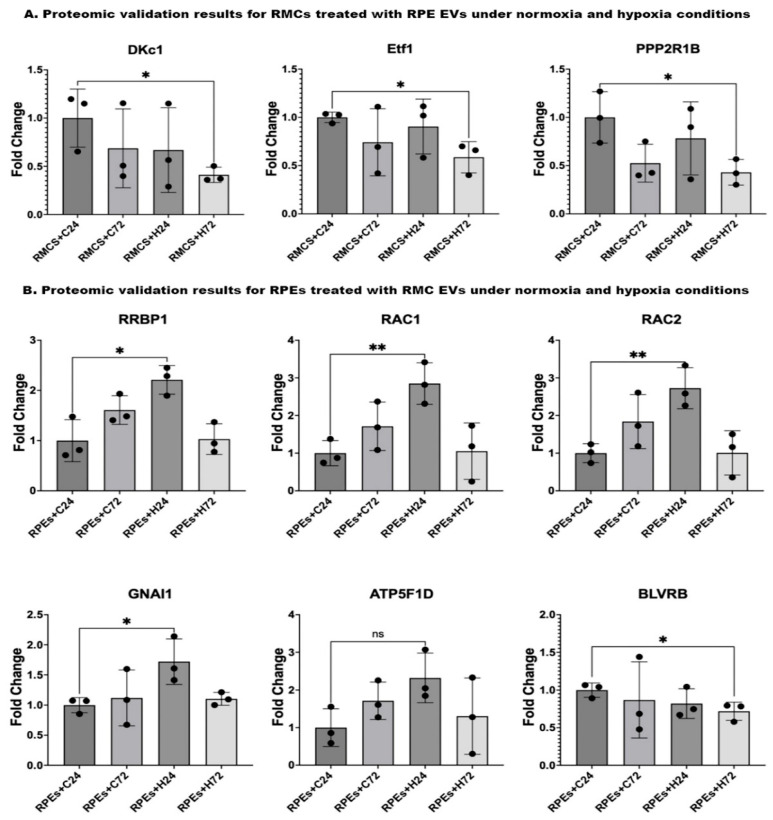
Proteomic validation of both RMCs and RPEs co-cultured with EVs under normoxic and hypoxic conditions using the peptide count from the proteomic data. (**A**) Proteomic validation results for RMCs treated with RPE EVs shows a significant reduction in DKc1, Etf1, and PPP2R1B gene expressions in RMCs treated with RPE EVs after 72 h of hypoxia compared to those RMCs treated with RPE EVs under normoxia for 24 h. (**B**) Proteomic validation results for RPEs treated with RMC EVs: Comparing the gene expressions in RPEs treated with RMC EVs after 24 h of hypoxia, those RPEs treated with RMC EVs under normoxia for 24 h showed a significant elevation in RRBP1, RAC1, RAC2, and GNAI1. On the other hand, there was a non-significant rise in ATP5F1D gene expression. BLVRB gene expression was significantly lower in RPEs treated with RMC EVs after 72 h of hypoxia when compared to RPEs treated with RMC EVs under normoxia for 24 h. Data represent individual data points with means ± SEM; *n* = 3; * *p* < 0.05; ** *p* < 0.01; ns: non-significant.

The global proteomic shifts observed in volcano plots (Figure 4 and Figure 6) provided a broad perspective on EV-induced changes. However, to enhance interpretability and focus on robust findings, we included only statistically validated proteins in Figure 7. While volcano plots offer a valuable global view of proteomic changes, not all proteins initially highlighted meet statistical significance upon direct comparison between experimental groups. This is an expected characteristic of large-scale omics datasets. Therefore, to ensure rigor and reproducibility, we performed focused post hoc analyses to identify and report only those proteins with statistically significant differences, as detailed in Figure 7.

Upon validating the data, there was a significant reduction in the levels of DKc1, ETF1, and PPP2R1B in RMCs co-cultured with hypoxic RPE EVs for 72 h when compared to the control group (Figure 7A). This finding aligns with the previously mentioned adaptive trials of RMCs to maintain their function as normally as possible. DKc1, which is known to play an active role in telomerase stabilization and maintenance, as well as the recognition of small nucleolar RNA ribonucleoproteins (snoRNPs). Its reduction in the studied cells may lead to impaired RNA processing, affect telomere stability, and facilitate the apoptosis of the cells [40]. DKC1 dysfunction leads to telomere shortening, which causes premature cellular apoptosis. Some studies in telomeropathy models suggest that retinal degeneration may occur secondary to telomerase dysfunction [41,42]. Ischemia induces a global shutdown of translation, but some stress-responsive proteins (e.g., HIF-1α, VEGF) need to be selectively translated [43]. DKC1 is part of the H/ACA ribonucleoprotein complex involved in rRNA modification (pseudouridylation) and stabilization the of various RNAs. Disruption of DKC1 function could impair the stress-adaptive translational landscape, leading to inefficient stress responses in ischemic tissues [44].

The ETF1 protein plays an essential role in directing the termination of mRNA translation by recognizing the stop codons. It has been linked to peptide chain elongation and nervous system development pathways. It is also involved in the regulation of apoptosis [45]. Hypoxic stress (e.g., in DR) triggers translational reprogramming [46]. Translation termination factors like ETF1 may be modulated under hypoxia to selectively translate survival or angiogenic proteins (e.g., VEGF). Dysregulation could contribute to abnormal angiogenesis or neuronal cell death in ischemic retinal diseases [47]. Translation termination factors like ETF1 are tightly linked to the quality control of newly synthesized proteins [48]. In retinal cells, particularly the RPE and Müller glia, this balance is critical. Disruption may exacerbate oxidative stress, promote inflammation, or lead to the cell death seen in retinal hypoxic conditions (e.g., DR, retinal vein occlusion) [49].

PPP2R1B is a group of enzymes that catalyze the removal of phosphate groups from serine and/or threonine residues through the hydrolysis of phosphoric acid monoesters. They oppose the action of kinases and phosphorylases and are involved in signal transduction [50]. Disruption in PPP2R1B expression or function could alter PP2A activity, which regulates apoptosis by dephosphorylating pro-apoptotic and anti-apoptotic proteins (e.g., Bad, Bcl-2, p53), contributing to excessive or impaired cell death in the retina [51,52]. PP2A negatively regulates inflammatory signaling pathways, including NF-κB, MAPK, and STAT3 [53]. In retinal glial cells (Müller glia, microglia), these pathways are activated during injury or stress [54]. A deficit in PPP2R1B might enhance inflammatory responses, exacerbating gliosis and retinal damage in diseases like DR or retinal ischemia.

In the current work, we assessed the barrier integrity in the RPE after culturing with RMC EVs under normoxic and hypoxic conditions (Figure 5). The resistance showed a dramatic decline over time among the hypoxic-EV-treated groups, but there was no significant change among the normoxic-EV-treated groups. The BRB consists of inner and outer components and plays an important role in the homeostatic regulation of the retinal microenvironment. The outer BRB (oBRB) is formed by tight junctions (TJ) between cells of the retinal pigment epithelium (RPE) [55].

In a hypoxic adult rat model in which a severe disruption of the iBRB is induced by two hours of hypoxia, no disruption of the oBRB was seen. The intercellular spaces between RPE cells were widened, but the TJs remained intact and prevented the impact of the systemically administered Horseradish peroxidase (HRP). Findings suggest that the oBRB is highly resistant to hypoxic damage, and the disruption of the oBRB, when it occurs, involves factors other than hypoxia, such as oxidative stress and the generation of ROS [56].

The present study showed the dominance of fatty acid metabolic pathways, together with ER-related and intracellular vesicular transport in RPE cells cultured with 24 h hypoxia RMC EVs (Figure 6d). These findings closely resemble the typical efficient metabolic function of the RPE, which plays a key role in supplying energy to retinal cells and preserving photoreceptor structure and function. The RPE primarily relies on substrates such as lactate, proline, and fatty acids for mitochondrial respiration, with ATP production mainly driven by the oxidative phosphorylation of lactate. Recent research has highlighted proline as the dominant metabolic substrate used by the RPE, which is synthesized within the cells from ornithine through the action of ornithine aminotransferase (OAT) [57].

RPE cells transport lipids to fuel photoreceptors, so normal lipid metabolism in the RPE is important for photoreceptor survival and function. Normal lipid metabolism is important for maintaining RPE mitochondrial metabolism. Fatty acids from shed photoreceptor outer segments are metabolized in the mitochondria of the RPE by β-oxidation to produce NADH, FADH2, and acetyl coenzyme A [58].

RMC EVs after 72 h of hypoxia have shifted the proteomic pathways of the co-cultured RPE (Figure 6e). The enhanced pathways included increased cytoplasmic ribosomes and function, increased intermediate filament expression, like the keratin filament, and most notably, the biosynthesis of Ubiquinone and other terpenoid-quinone compounds. Ubiquinone (UQ), also known as coenzyme Q (CoQ), is a redox-active lipid that is present in all cellular membranes, where it acts in a range of biological activities, mostly to enhance ATP production and minimize reactive oxygen species production [59].

Upon validating the data, the levels of RRBP1, RAC1 &2, and GANI1 were significantly elevated, but ATP5P1D was non-significantly increased in the RPE cultured with hypoxic RMC EVs for 24 h compared to the control group. BLVRB was significantly reduced in the RPE cultured with hypoxic RMC EVs for 72 h compared to the control group (Figure 7B).

Aligning with the proteomic findings, RRBP1 (also referred to as p180) is a membrane-bound protein found in the endoplasmic reticulum that enhances the association of certain mRNAs with the endoplasmic reticulum. It plays a role in ER morphology, ER proliferation, secretory pathways, secretory cell differentiation, and the mediation of ER–microtubule interactions [60]. Hypoxia impairs oxygen-dependent protein folding in the ER, causing ER stress and activating the unfolded protein response (UPR) [61]. RRBP1 contributes to mRNA anchoring and protein translation on the rough ER. RRBP1 dysregulation may disrupt protein quality control, increase misfolded protein accumulation, and lead to apoptosis or inflammatory signaling [62].

RAC1 and RAC2 are small GTPases of the Rho family that regulate critical cellular functions such as cytoskeletal remodeling, oxidative stress signaling, cell survival, and inflammation. Both play overlapping yet distinct roles depending on cell type and context. While RAC1 is ubiquitously expressed, RAC2 is primarily found in hematopoietic and immune cells [63]. In the context of retinal hypoxia as seen in diseases like DR, retinal vein occlusion (RVO), and AMD, RAC1 and RAC2 are implicated through several mechanistically relevant pathways [64,65,66]. RAC1 activates NADPH oxidase, particularly NOX2, leading to the generation of ROS [67]. In hypoxic retinal tissue, this contributes to endothelial dysfunction, blood–retinal barrier (BRB) breakdown, and oxidative damage to photoreceptors and RPE cells [68,69]. In Müller glial cells, Rac1 contributed to the crosstalk between different signaling pathways activated in Müller glia after injury [70]. Rac1 is responsible for activating the expression of hypoxia-induced HIF-1α. Upregulation of VEGF in hypoxic RPE cells, mediated by HIF-1, is responsible for choroidal neovascularization. Zhang et al. found that the levels of HIF-1α and Rac1 proteins significantly increase in a time-dependent manner under hypoxia. The expression of HIF-1α protein reaches the maximum at 8 h, and Rac1 protein reaches the maximum at 4 h, which starts to decrease after that [64].

GNAI1 is a G-protein α-subunit involved in inhibitory signaling pathways, notably those mediated by G-protein-coupled receptors (GPCRs). Direct studies linking GNAI1 to retinal diseases are limited, but it has been linked to colitis, colon cancer, seizures, hypotonia, and neurodevelopmental disorders [71,72,73]. In colorectal cancer models, exosomal miR-320d has been shown to downregulate GNAI1 expression in vascular endothelial cells. This downregulation leads to the activation of the JAK2/STAT3 signaling pathway and an increase in VEGFA expression, promoting angiogenesis [74]. GNAI1’s involvement in inhibitory signaling pathways suggests that it could modulate responses to hypoxia, possibly by influencing angiogenic signaling or interacting with hypoxia-responsive transcription factors.

ATP5F1D (formerly known as ATP5D) encodes the delta subunit of mitochondrial ATP synthase (Complex V), a critical enzyme in oxidative phosphorylation that is responsible for ATP production. While direct studies linking ATP5F1D to retinal hypoxia are limited, emerging research highlights the broader role of mitochondrial ATP synthase subunits in retinal energy metabolism and hypoxia-induced pathologies [75]. Under hypoxic conditions, such as diabetic retinopathy, AMD, and oxygen-induced retinopathy (OIR), mitochondrial functions are compromised, including altered ATP Synthase activity that leads to decreased ATP production, exacerbating energy deficits in retinal cells [76,77,78].

BLVRB (Biliverdin Reductase B) is an enzyme that catalyzes the conversion of biliverdin to bilirubin. It is a potent antioxidant that enables biliverdin reductase [NAD(P)+] activity, peptidyl-cysteine S-nitrosylase activity, and riboflavin reductase (NADPH) activity. It is in cytosol, nucleoplasm, and the plasma membrane. It acts as a regulator of hematopoiesis, intermediary metabolism (glutaminolysis, glycolysis, the TCA, and the pentose phosphate pathway) [79]. Studies on BLVRB in retinal hypoxia are scarce; the enzyme’s antioxidant role suggests that it could mitigate oxidative damage in hypoxic retinal conditions.

## 5. Conclusions

Our study highlights the critical role of EV-mediated crosstalk between RPE and Müller cells in adapting to hypoxia. Short-term hypoxia induces a protective metabolic shift in Müller cells, supporting neurotransmitter recycling and maintaining cellular function, while prolonged hypoxia leads to metabolic reprogramming geared toward enhancing mitochondrial respiration and ATP production. Concurrently, hypoxic RPE cells exhibit alterations in lipid metabolism, intracellular vesicular transport, and the biosynthesis of mitochondrial co-factors such as ubiquinone, all of which contribute to sustaining retinal energy demands and minimizing oxidative stress.

Proteomic analysis revealed the time-dependent modulation of key molecular pathways and proteins, including reduced levels of DKc1, ETF1, and PPP2R1B, implicating changes in RNA processing, translation termination, and signal transduction, as well as the elevated expression of proteins like RRBP1, RAC1/2, and GNAI1 that support endoplasmic reticulum function, cytoskeletal dynamics, and neurodevelopmental signaling. The changes in barrier integrity observed in the oBRB further emphasize the selective vulnerability and resilience of retinal components to hypoxic stress.

Together, these findings underscore the retina’s intricate response to hypoxia, involving coordinated metabolic and molecular adaptations in Müller and RPE cells. EV-mediated intercellular communication emerges as a crucial mechanism for maintaining retinal homeostasis and function under adverse conditions, offering potential therapeutic targets for preventing or mitigating hypoxia-related retinal degeneration.

## Data Availability

The original contributions presented in this study are included in the article/Supplementary Materials. Further inquiries can be directed to the corresponding author(s).

## Supplementary Materials

The following supporting information can be downloaded at: https://www.mdpi.com/article/10.3390/biology14081014/s1.

## Abbreviations

The following abbreviations are used in this manuscript:

DR	Diabetic retinopathy
AMD	Age-related macular retinopathy
GFAP	Glial fibrillary acidic protein
RPE	Retinal pigment epithelium
EVs	Extracellular vesicles
oBRB	Outer blood–retinal barrier
PEDF	Pigment epithelial-derived factor
VEGF	Vascular epithelial growth factor
HIF-1	Hypoxia-inducible factor 1
ROS	Reactive oxygen species
RP	Retinitis pigmentosa
SD	Stargardt disease
FBS	Fetal bovine serum
PBS	Phosphate-buffered saline
TEM	Transmission electron microscope
ECIS	Electric cell-substrate impedance sensing method
TER	Transcellular electrical resistance
MF	Molecular function
CC	Cellular component
BP	Biological process
KEGG	Kyoto Encyclopedia of Genes and Genomes
ER	Endoplasmic reticulum
PVR	Proliferative vitreoretinopathy
TCA	Tricarboxylic acid cycle
DKC1	Dyskerin Pseudouridine Synthase 1
snoRNPs	small nucleolar RNA ribonucleoproteins
ETF1	Eukaryotic Translation Termination Factor 1
PPP2R1B	Protein Ser/Thr phosphatases
TJ	Tight junctions
HRP	Horseradish peroxidase
OAT	Ornithine aminotransferase
UQ	Ubiquinone
CoQ	coenzyme Q
RRBP1	Ribosome-binding protein 1
URP	Unfolded protein response
RVO	Retinal vein occlusion
GNAI1	Guanine Nucleotide-Binding Protein G(i) Subunit Alpha-1
GPCRs	G-protein-coupled receptors
ATP5F1D	delta subunit of mitochondrial ATP synthase (Complex V)
OIR	Oxygen-induced retinopathy
BLVRB	Biliverdin Reductase B
